# Association between non-high-density lipoprotein cholesterol-to-high-density lipoprotein cholesterol ratio and the risk of gestational diabetes: a retrospective study

**DOI:** 10.3389/fnut.2025.1617225

**Published:** 2025-07-03

**Authors:** Guohua Li, Dongxing Su, Wei Huang, Xuehua Wu, Yanzhu Huang

**Affiliations:** ^1^Clinical Laboratory, Jinjiang Municipal Hospital, Quanzhou, China; ^2^Obstetrics and Gynecology Department, Jinjiang Municipal Hospital, Quanzhou, China

**Keywords:** association, ROC curve, RCS curve, non-HDL-to-HDL cholesterol ratio, gestational diabetes mellitus

## Abstract

**Background:**

Previous studies have shown an association between non-high-density lipoprotein cholesterol (non-HDL-C)-to-HDL-C ratio (NHHR) and diabetes; however, its impact on pregnant women remains unclear. This study aims to explore the association between NHHR in early to mid-pregnancy and the incidence of gestational diabetes mellitus (GDM).

**Methods:**

This study retrospectively collected and analyzed prenatal examination data from pregnant women. Variables were selected using least absolute shrinkage and selection operator (LASSO) regression and multivariable logistic regression, and the association between NHHR and GDM incidence was assessed through sensitivity and subgroup analyses. Propensity score matching (PSM) was applied to reduce selection bias between groups. Additionally, receiver operating characteristic (ROC) analysis was performed to assess the predictive accuracy of NHHR for GDM.

**Results:**

The study included 572 pregnant women aged 20–44 years, with a mean age of 31.35 years (standard deviation: 4.02). LASSO and multivariable logistic regression analyses identified NHHR as an independent risk factor for GDM. Despite adjusting for group differences using PSM, NHHR values remained significantly different between groups (*p* = 0.009). The predictive accuracy of NHHR for GDM was 0.625 [95% confidence interval (CI): 0.570–0.679]. Multivariable logistic regression analysis revealed a significant positive association between NHHR and GDM (odds ratio: 2.25; 95% CI: 1.27–3.98). Furthermore, the association between NHHR and GDM appeared linear (P for non-linearity > 0.05), and the positive correlation remained consistent across most subgroups.

**Conclusion:**

This study suggests that an elevated NHHR is associated with an increased risk of GDM. Early measurement of NHHR could help identify women at risk for GDM, potentially enabling timely interventions to improve pregnancy outcomes.

## Introduction

Gestational diabetes mellitus (GDM) is a metabolic disorder diagnosed during pregnancy and is one of the most common pregnancy-associated complications (1, 2). Studies suggest that the occurrence of GDM is influenced by both environmental and genetic factors (3). With global economic growth and improved living standards, the prevalence of GDM has been increasing. In 2021, the International Diabetes Federation reported a global prevalence of GDM at 16.7% (4). Additionally, the incidence of GDM is projected to continue rising due to aging populations, increasing obesity rates, and more sedentary lifestyles (5, 6). A 2-year prospective study involving 1,035 women with GDM found that, although most patients return to normal blood glucose levels postpartum, approximately 35% exhibit impaired glucose tolerance within the first 2 months postpartum (7). Furthermore, a multicenter, biracial cohort study in the United States revealed that women with a history of GDM have a significantly higher risk of developing type 2 diabetes mellitus (T2DM) later in life, highlighting the long-term impact of GDM on diabetes risk (8). GDM is associated with a range of adverse perinatal outcomes, including macrosomia, preterm birth, birth trauma, neonatal hypoglycemia, respiratory distress, pregnancy-induced hypertension, preeclampsia, and cesarean section (9). Therefore, identifying risk factors that predict or are associated with GDM is crucial for developing effective prevention strategies.

Recently, the relationship between lipid metabolism and various metabolic disorders, particularly diabetes, has garnered significant attention (10). A Canadian observational study found that non-high-density lipoprotein cholesterol (non-HDL-C) is more strongly associated with T2DM than low-density lipoprotein cholesterol (LDL-C) (11). Using receiver operating characteristic (ROC) curve analysis, Xie et al. (12) demonstrated that incorporating cholesterol levels into risk prediction models could enhance the accuracy of diabetes risk prediction by 12.66%. These findings indicate that atherosclerotic lipid abnormalities serve as an independent risk factor for diabetes. The non-HDL-C-to-HDL-C ratio (NHHR) is an emerging composite index for evaluating lipid status in atherosclerosis (13). Recent studies indicate that NHHR provides superior predictive and diagnostic results in evaluating the risks of atherosclerosis, chronic kidney disease, non-alcoholic fatty liver disease, metabolic syndrome, and insulin resistance compared to conventional lipid metrics (14). Compared to single lipid parameters, NHHR simultaneously integrates components related to both lipid clearance and deposition, providing a more comprehensive assessment of the dynamic state of lipid metabolism. Yet, to our knowledge, no studies have examined the association between NHHR and GDM.

This study aims to investigate the association between NHHR in early to mid-pregnancy and the incidence of GDM through a retrospective analysis of routine prenatal and clinical data and to further assess the clinical utility of NHHR as an early predictive marker for GDM risk.

## Methods

### Study participants

**Figure 1:**
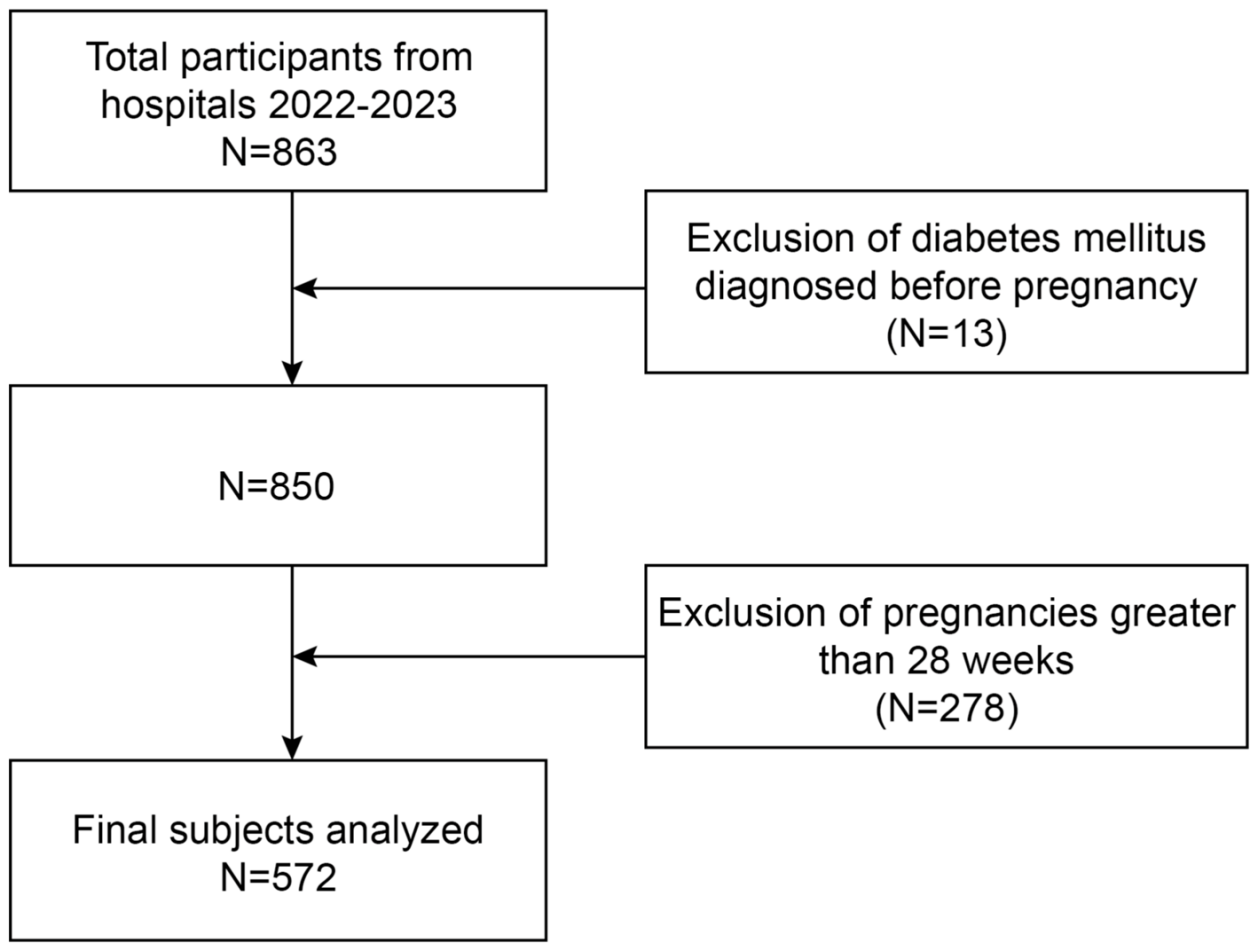
Flowchart of participation rule selection.

This study utilized the hospital’s electronic medical record system to collect routine prenatal examination data electronically from Jinjiang City Hospital between 1 January 2022 and 31 December 2023. All patients’ personal information was protected under the supervision of the ethics committee. The inclusion criteria were as follows: (1) women who were naturally conceived and pregnant and (2) women who had prenatal checkups at our hospital. The exclusion criteria included were as follows: (1) women diagnosed with or being treated for diabetes prior to pregnancy; (2) women beyond 28 weeks of gestation; (3) minors (under 18 years old); (4) women with cardiovascular diseases, liver diseases, renal insufficiency, or other chronic conditions, such as hypertension, dyslipidemia, thyroid disorders, and polycystic ovary syndrome (PCOS); (5) women using hormones that impact lipid and glucose metabolism, such as glucocorticoids or immunosuppressants; and (6) multiple pregnancies (≥2 fetuses) and abnormal pregnancies that did not result in successful delivery were excluded (Figure 1). The study protocol was approved by the Ethics Committee of Jinjiang Municipal Hospital (jjsyyll-2024-025). Due to the retrospective nature of the study, the requirement for informed consent was waived.

### Diagnosis of GDM

The study followed the International Association of Diabetes and Pregnancy Study Groups’ criteria for diagnosing GDM. Pregnant women were tested between 24 and 28 weeks of gestation with a 75 g oral glucose tolerance test. The diagnostic test for GDM included three blood glucose measurements taken over a 2-h period: fasting blood glucose levels of ≥5.1 mmol/L, 1-h post-load glucose levels of ≥10.0 mmol/L, and 2-h post-load glucose levels of ≥8.5 mmol/L. A diagnosis of GDM was confirmed if any one of these thresholds was exceeded.

### Calculation of NHHR

NHHR, a newly developed composite index for assessing atherosclerotic lipid status, is calculated as the difference between total cholesterol and HDL-C, divided by HDL-C (15, 16). In this study, NHHR values obtained during early and mid-pregnancy physical examinations were used as exposure variables.

### Covariates

Based on a literature review, this study collected clinical data from routine prenatal examinations of women in early to mid-pregnancy. The data included the following laboratory parameters: aspartate aminotransferase (AST), alanine aminotransferase (ALT), total cholesterol, albumin, creatinine, blood urea nitrogen, D-dimer, prothrombin time (PT), international normalized ratio (INR), activated partial thromboplastin time (APTT), thrombin time (TT), fibrinogen, free thyroxine (FT4), free triiodothyronine (FT3), thyroid-stimulating hormone (TSH), anti-thyroid peroxidase, anti-thyroglobulin, TSH receptor antibodies, leukocytes, hemoglobin, platelets, gamma-glutamyl transferase (GGT), triglycerides, and HDL-C. Additional general information, including age, gestational week, number of pregnancies, parity, and assisted reproduction status, was also collected.

### Statistical analysis

Statistical analysis was conducted using R version 3.6.4 and SPSS version 25.0. Key features related to GDM were identified using the least absolute shrinkage and selection operator (LASSO). The eight features selected at the minimal lambda value were incorporated into a multivariate regression analysis to identify independent risk factors for GDM. ROC curves were used to assess the discriminative ability of the three independent risk factors, with the area under the ROC curve (AUC) serving as the metric. On this basis, we selected the cutoff value corresponding to the maximum value of the Youden index as the optimal threshold for further analysis. To minimize selection bias, we applied 1:1 nearest-neighbor propensity score matching (PSM). To further validate the relationship between NHHR and GDM, we constructed three logistic regression models. Model 1 included no covariates. Model 2 adjusted for eight covariates identified by LASSO regression: age, atherosclerosis index, APTT, white blood cells (WBCs), FT3, triglyceride, gamma-glutamyltransferase (GGT), and platelet. Model 3 adjusted for all covariates. NHHR was also transformed from a continuous variable to a categorical variable for sensitivity analysis. Additionally, restricted cubic spline (RCS) plots were used to explore the non-linear relationship between NHHR and GDM. Finally, we analyzed the relationship between NHHR and GDM in subgroups stratified by age, gestational week, number of pregnancies, and parity, with interaction tests to examine the consistency of this association across subgroups.

## Results

### Baseline information

This study included 572 women who met the predefined inclusion and exclusion criteria, with a mean age of 31.35 ± 4.02 years. Table 1 presents a comparison of clinical data across the groups. No statistically significant differences were observed between the groups regarding gestational weeks, ALT, AST, albumin, total cholesterol, urea nitrogen, creatinine, D-dimer, INR, PT, fibrinogen, TT, FT3, FT4, TSH, anti-thyroglobulin, anti-thyroid peroxidase, TSH receptor antibodies, number of deliveries, and assisted reproduction techniques (all *p* > 0.05). However, compared to the non-GDM group, women in the GDM group were significantly older. They had higher levels of leukocyte count, hemoglobin, platelets, GGT, urea nitrogen, triglycerides, and NHHR, while APTT levels were significantly lower (*p* < 0.05).

**Table 1:** Clinical baseline table of participants.

Variables	Before PSM			After PSM		
	Non-GDM	GDM	*p*-value	Non-GDM	GDM	*p*-value
	*N* = 121	*N* = 451		*N* = 115	*N* = 115	
Age	28.97 ± 3.43	31.99 ± 3.93	**<0.001**	29.27 ± 3.21	29.45 ± 3.63	0.687
Week of pregnancy	17.24 ± 6.59	16.80 ± 7.12	0.452	17.32 ± 6.53	17.98 ± 7.28	0.469
Leucocyte	8.85 ± 1.97	9.40 ± 2.10	**0.009**	8.89 ± 1.97	9.21 ± 2.07	0.225
Hemoglobin	122.44 ± 10.13	129.12 ± 74.52	**0.019**	122.45 ± 10.18	123.139 ± 11.40	0.630
Blood platelet	234.03 ± 45.46	250.80 ± 53.23	**<0.001**	234.95 ± 46.06	234.09 ± 51.25	0.894
Glutamine aminotransferase	13.36 ± 9.08	15.60 ± 14.11	0.06	13.34 ± 8.71	13.98 ± 13.31	0.662
Glutamic transaminase	15.44 ± 4.94	15.61 ± 8.74	0.125	15.35 ± 4.59	14.99 ± 8.00	0.675
Albumin	41.88 ± 3.22	42.39 ± 19.52	0.741	41.85 ± 3.24	41.25 ± 4.85	0.267
Total cholesterol	6.56 ± 2.93	6.16 ± 3.43	0.069	6.50 ± 2.89	6.02 ± 5.05	0.378
Gamma-glutamyltransferase	11.64 ± 9.05	15.17 ± 13.46	**<0.001**	11.79 ± 9.22	12.24 ± 6.99	0.677
Urea nitrogen	2.75 ± 0.68	3.32 ± 12.04	0.411	2.75 ± 0.70	2.77 ± 0.73	0.802
Creatinine	44.91 ± 7.97	44.28 ± 8.43	0.306	44.89 ± 8.11	44.84 ± 11.78	0.967
Uric acid	223.74 ± 41.16	243.40 ± 117.35	**0.012**	223.77 ± 40.99	228.67 ± 61.22	0.477
Triglyceride	1.38 ± 0.43	1.63 ± 0.68	**0.002**	1.40 ± 0.42	1.48 ± 0.67	0.258
NHHR	1.57 ± 0.43	1.80 ± 0.57	**<0.001**	1.57 ± 0.43	1.73 ± 0.49	**0.009**
D-dimer	0.75 ± 0.60	0.82 ± 1.35	0.268	0.76 ± 0.61	0.90 ± 1.28	0.291
INR	0.96 ± 0.05	0.95 ± 0.06	0.792	0.96 ± 0.05	2.12 ± 12.41	0.317
PT	11.30 ± 0.61	11.24 ± 0.86	0.492	11.29 ± 0.60	11.19 ± 1.17	0.424
APTT	30.13 ± 18.86	27.82 ± 4.88	**0.026**	28.28 ± 2.37	28.63 ± 8.73	0.678
Fib	3.80 ± 0.49	3.83 ± 0.84	0.774	3.80 ± 0.50	3.85 ± 1.24	0.655
TT	15.53 ± 1.10	15.39 ± 1.54	0.765	15.50 ± 1.1	15.34 ± 1.75	0.399
FT3	4.75 ± 0.59	4.84 ± 0.72	0.415	4.75 ± 0.61	4.70 ± 0.58	0.462
FT4	15.83 ± 2.12	16.10 ± 7.12	0.506	15.81 ± 2.15	15.39 ± 2.48	0.173
TSH	1.41 ± 1.02	1.44 ± 0.86	0.348	1.41 ± 1.04	1.48 ± 0.79	0.562
Anti-TG	19.76 ± 28.80	22.55 ± 43.17	0.063	19.97 ± 29.53	24.71 ± 60.23	0.450
Anti-TPO	16.05 ± 53.77	16.96 ± 48.88	0.33	16.40 ± 55.14	13.16 ± 23.76	0.563
A-TSHR	0.89 ± 0.41	0.92 ± 0.75	0.13	0.89 ± 0.42	0.893 ± 0.684	0.968
Number of pregnancies			**0.01**			1.000
<3	103 (85.12%)	335 (74.28%)		97 (84.35)	96 (83.48)	
≥3	18 (14.88%)	116 (25.72%)		18 (15.65)	19 (16.52)	
Parities			0.27			0.719
<3	116 (95.87%)	420 (93.13%)		110 (95.65)	112 (97.39)	
≥3	5 (4.13%)	31 (6.87%)		5 (4.35)	3 (2.61)	
Assisted reproduction			0.052			0.719
0	118 (97.52%)	418 (92.68%)		112 (97.39)	110 (95.65)	
1	3 (2.48%)	33 (7.32%)		3 (2.61)	5 (4.35)	

anti-TPO, anti-thyroid peroxidase antibody; TSHR, thyroid stimulating hormone receptor. Bold indicates that the variables are statistically different between the two groups.

### Screening for risk factors for GDM

**Figure 2:**
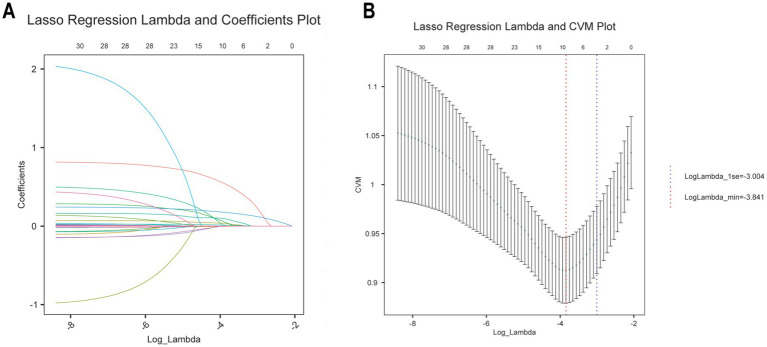
Selection of demographic and clinical characteristics using LASSO regression. **(A)** LASSO regression lambda value mean square error plot. **(B)** Plot of coefficients of LASSO regression with lambda values.

To comprehensively evaluate the risk factors associated with GDM, LASSO regression was initially used for variable selection. The optimal parameter (lambda) for the LASSO model was determined using 10-fold cross-validation. Vertical dashed lines in the plot indicate the optimal model selection criteria and one standard error from it. At the minimum lambda value, eight variables with non-zero coefficients were identified, including age, NHHR, APTT, WBC count, FT3, triglycerides, GGT, and platelets (Figures 2A,B). The LASSO regression coefficients for these key variables related to GDM are detailed in Supplementary Table 1.

### Independent risk factors for GDM

**Figure 3:**
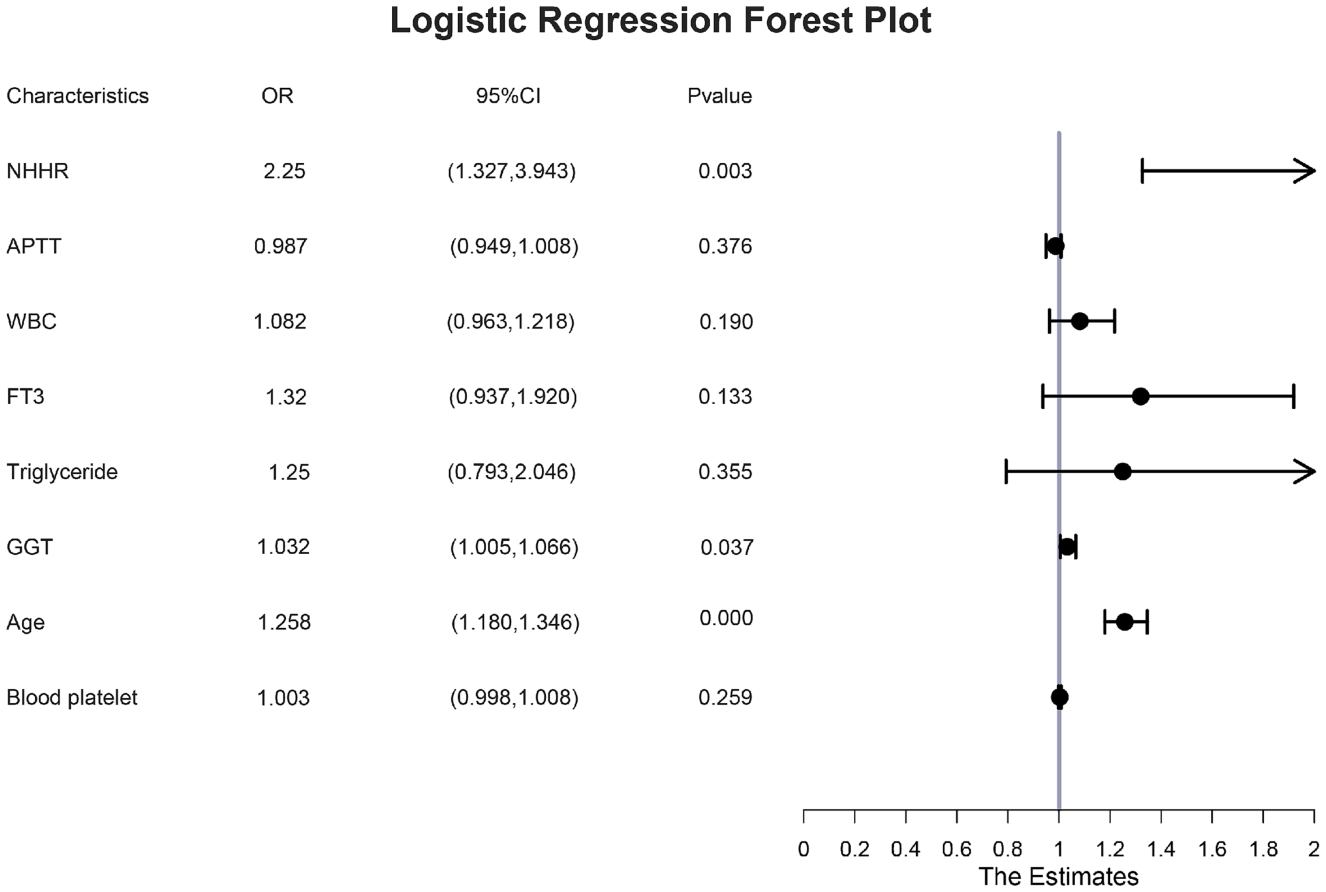
Multi-factor logistic regression forest plot.

The study conducted a multivariate logistic regression analysis to identify independent risk factors for GDM. The eight factors identified through LASSO regression were tested for multicollinearity, with no significant collinearity detected (variance inflation factor < 2), as detailed in Supplementary Table 2. These variables were then incorporated into a multivariate logistic regression model. The analysis confirmed that age [odds ratio (OR): 1.258; 95% confidence interval (CI): 1.180–1.346], NHHR (OR: 2.25; 95%CI: 1.337–3.943), and GGT (OR: 1.032; 95%CI: 1.005–1.066) were independent risk factors for GDM (Figure 3).

### NHHR for predicting the incidence of GDM

To evaluate the predictive effectiveness of NHHR for the incidence of GDM, the study constructed ROC curves based on the multivariate logistic regression analysis, assessing the predictive performance of age, NHHR, and GGT. The results showed that the AUC for age, NHHR, and GGT was 0.722 (95% CI: 0.672–0.772), 0.625 (95% CI: 0.570–0.679), and 0.626 (95% CI: 0.570–0.683), respectively, all demonstrating potential predictive value for GDM. The predictive roles of age and GGT are well established, given the extensive research literature on their association with GDM (17–19). Therefore, this study focused on exploring the association between NHHR and GDM. The results showed a sensitivity of 45.23%, a specificity of 73.55%, a positive likelihood ratio (positive-LR) of 1.710, and a negative likelihood ratio (negative-LR) of 0.745.

### PSM

**Figure 4:**
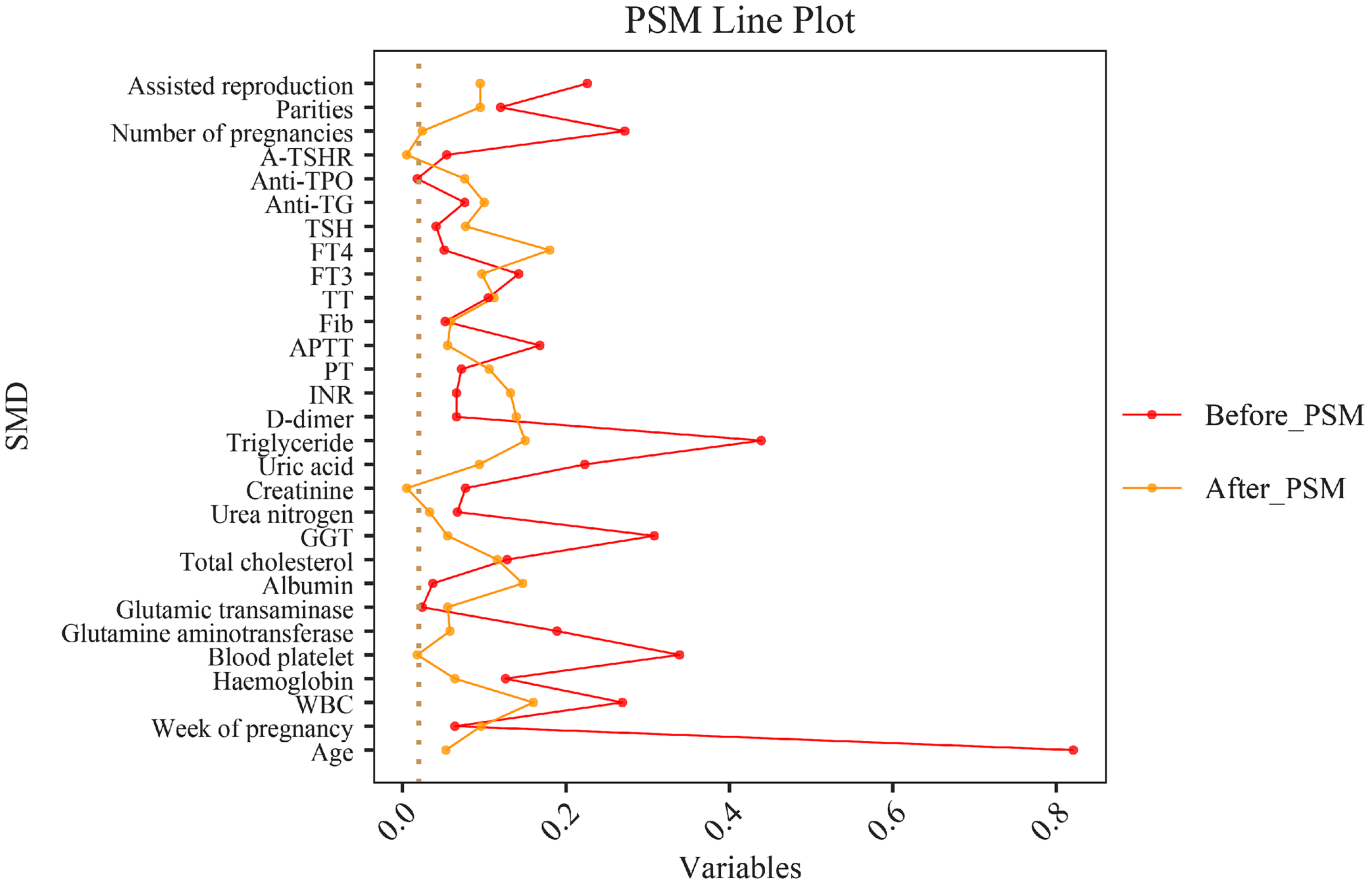
Standardized mean difference (SMD) before and after propensity score matching.

To reduce selection bias and enhance the reliability of studies on the correlation between NHHR and GDM, patients in this study were divided into two groups based on the presence or absence of GDM and matched using a 1:1 PSM method. After PSM, the differences between the GDM and non-GDM groups in other covariates did not reach statistical significance (Table 1). Additionally, Figure 4 illustrates the standardized mean differences before and after PSM. The findings revealed that the NHHR levels were significantly higher in the matched GDM group compared to the non-GDM group (*p* = 0.009), supporting the hypothesis of a potential association between NHHR levels and GDM.

### The link between NHHR and GDM

**Figure 5:**
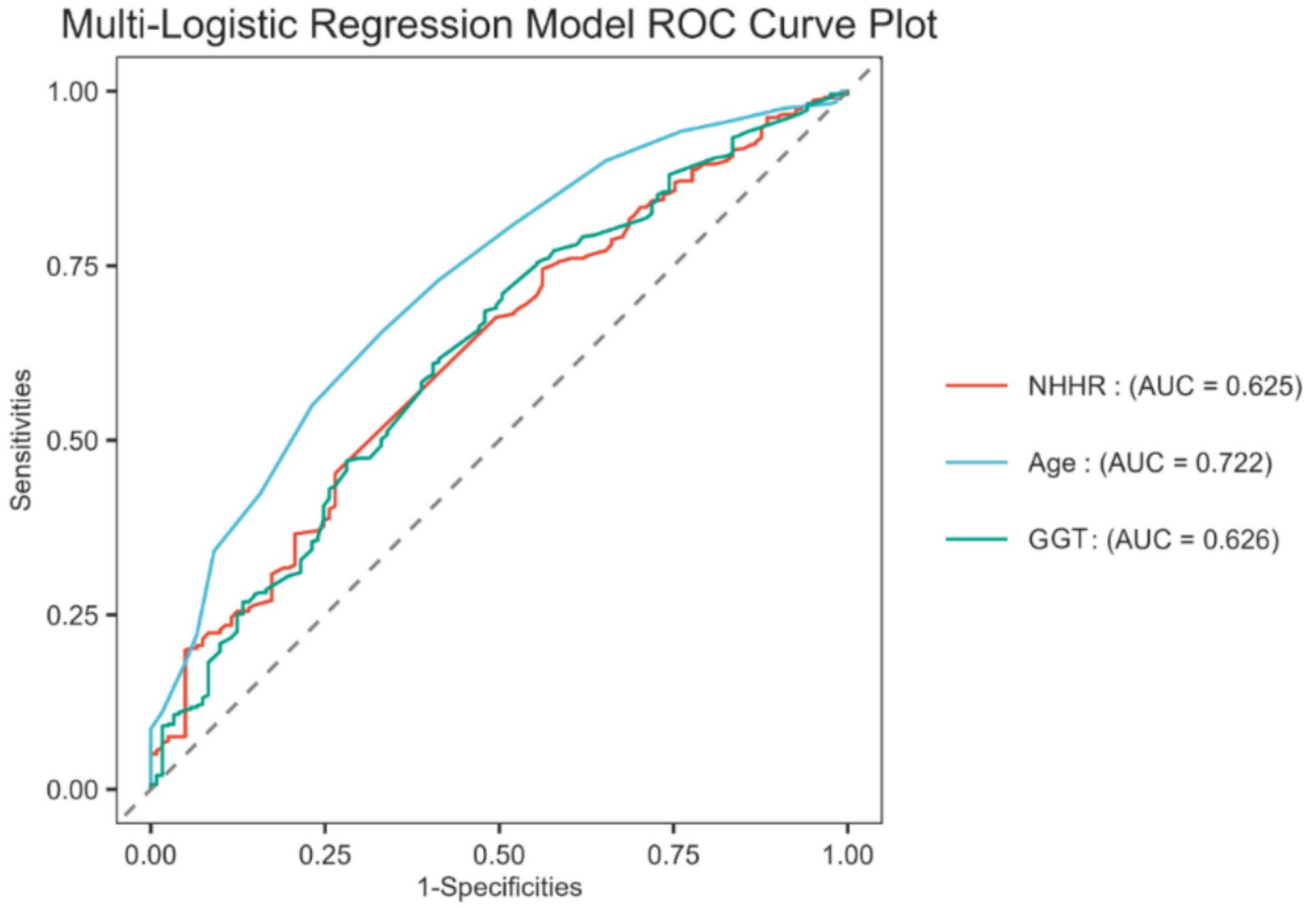
ROC curve used to predict GDM.

Table 2 presents the results of a multivariable logistic regression analysis conducted using three models, demonstrating a strong positive association between NHHR levels and the risk of developing GDM. This association was significant in the unadjusted model (Model 1: OR: 2.64; 95% CI: 1.65–4.21). Additionally, the positive correlation remained statistically significant in both the partially and fully adjusted models (Model 2: OR: 2.25; 95% CI: 1.21–3.12; Model 3: OR: 2.25; 95% CI: 1.27–3.98). Analyzing the ROC curve for NHHR and GDM (Figure 5), the optimal cutoff value was determined to be NHHR = 1.64. Subsequently, NHHR was transformed from a continuous variable into a categorical variable for further analysis, and the positive association between NHHR and GDM remained consistent across all models (Model 1: OR: 2.12; 95% CI: 1.41–3.19; Model 2: OR: 1.94; 95% CI–1.17, 3.09; Model 3: OR: 2.08; 95% CI: 1.25–3.46).

**Table 2:** Association between NHHR and GDM.

NHHR	Crude model (Model 1)		Minimally adjusted model (Model 2)		Fully adjusted model (Model 3)	
	OR (95% CI)	*p*-value	OR (95% CI)	*p*-value	OR (95% CI)	*p*-value
Continuous	2.64 (1.65, 4.21)	<0.0001	2.25 (1.31, 3.87)	0.0034	2.25 (1.27, 3.98)	0.0054
Categories						
<1.64	Reference		Reference		Reference	
≥1.64	2.12 (1.41, 3.19)	0.0003	1.94 (1.21, 3.12)	0.0057	2.08 (1.25, 3.46)	0.0049

Model 1, No variables were adjusted. Model 2, Adjusted for age, APTT, WBCs, FT3, triglycerides, GGT, and platelets. Model 3, Adjusted for all covariates.

Furthermore, after adjusting for all confounders in Model 3, each unit increase in the NHHR ≥ 1.64 group was associated with a 2.08-fold increased risk of GDM compared to the NHHR <1.64 group (Model 3: OR: 2.08; 95% CI: 1.25–3.46).

### Dose–response relationship between NHHR and prevalence of GDM

**Figure 6:**
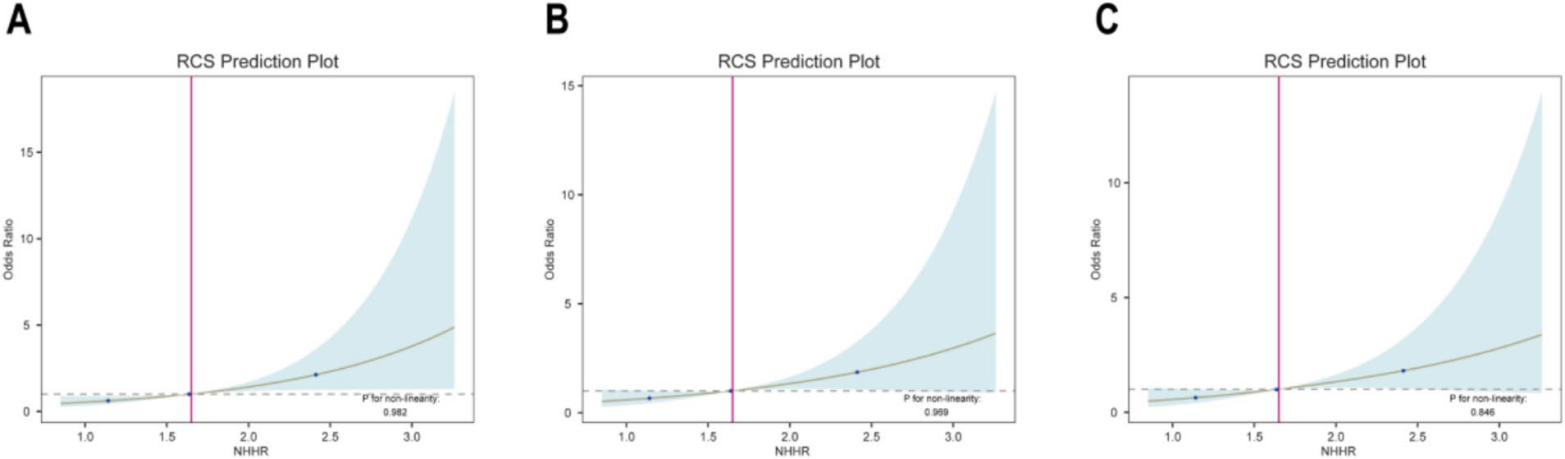
**(A-C)** Restricted cubic spline plot of the relationship between NHHR and GDM.

This study utilized RCS plots to examine the association between NHHR and the prevalence of GDM (Figure 6). Based on multivariate logistic regression models, three models were developed to adjust for confounders. The results from the RCS curves of all three models showed a significant linear positive association between NHHR and the prevalence of GDM (all P for non-linearity > 0.05). Therefore, the prevalence of GDM increased in direct proportion to higher NHHR levels.

### Subgroup analysis of the correlation between NHHR and GDM

Subgroup analyses were conducted to assess the consistency of the association between NHHR and GDM across different subgroups based on age, gestational week, number of pregnancies, and number of deliveries (Table 3). The results indicate that the positive correlation between NHHR and GDM persisted across all subgroups and remained statistically significant in women of childbearing age, those in mid-pregnancy, and those with fewer than three pregnancies or deliveries. Additionally, interaction tests showed no significant differences in the relationship between NHHR and GDM across the stratifications (P for interaction > 0.05), suggesting a positive and consistent correlation between NHHR and GDM in most populations.

**Table 3:** Subgroup analysis of the relationship between NHHR and GDM.

Variable	OR	95%CI	*p*-value	P for interaction
Age				0.7289
Non-advanced maternal age	1.96	(1.12, 3.42)	0.0180	
Advanced maternal age	2.77	(0.40, 19.10)	0.3019	
Week of pregnancy				0.2627
Early pregnancy	1.78	(0.87, 3.66)	0.1156	
Mid-pregnancy	3.29	(1.37, 7.87)	0.0075	
Number of pregnancies				0.8527
<3	2.29	(1.23, 4.26)	0.0087	
≥3	2.02	(0.60, 6.79)	0.2548	
Parities				0.6285
<3	2.19	(1.23, 3.91)	0.0079	
≥3	4.82	(0.14,170.70)	0.3872	

Pregnant women younger than 35 years of age are considered to have a non-advanced maternal age, whereas those aged 35 years and older are recognized as having an advanced maternal age. Gestational weeks less than 13 weeks are considered early pregnancy, and gestational weeks of 14 weeks 0 days to 27 weeks 6 days are considered to be mid-pregnancy.

## Discussion

This study investigated the association between NHHR levels during early to mid-pregnancy and the risk of GDM. We conducted a retrospective analysis of data from 572 pregnant women who underwent prenatal screening between January 2022 and December 2023. Using LASSO regression and multivariate regression analysis, we identified NHHR as an independent risk factor for GDM. The ROC curve analysis revealed an AUC of 0.625 for NHHR, confirming its potential as a predictive marker for GDM. Additionally, both multivariate logistic regression and RCS curve analysis demonstrated a stable positive correlation between NHHR and GDM. Subgroup analyses and interaction tests further validated the consistency of this positive relationship across different population groups. In summary, our study indicates that increased NHHR levels in early pregnancy to mid-pregnancy are associated with an increased risk of GDM and can serve as an independent predictor.

This study was designed to evaluate the relationship between NHHR and GDM during early and mid-pregnancy. Early identification of GDM risk factors can prompt pregnant women to adopt healthier lifestyle changes, which may help reduce the incidence of GDM. A growing body of evidence indicates that NHHR is a reliable indicator of risk for lipid-related diseases (20–22). While studies directly examining the relationship between NHHR and GDM are limited, numerous investigations have highlighted associations between GDM and various lipid-related factors (23, 24). Research has shown that changes in lipid levels during pregnancy, particularly alterations in lipoprotein quality and function, are directly linked to the development of GDM (25).

A cohort study of 10,234 pregnant women found a strong association between GDM and hyperlipidemia (OR: 1.80; 95% CI: 1.73–1.88) (26). Additionally, elevated levels of specific lipid markers, such as the triglyceride-bound glucose index, have been associated with reduced insulin sensitivity and an increased risk of GDM (27). Kim et al. recently reported that, in models adjusted for confounders, pregnant women with higher triglyceride-glucose indices in the first trimester were 4.9 and 5.3 times more likely to develop GDM and deliver large-for-gestational-age infants, respectively, compared to those in the lower and lowest triglyceride-glucose quartiles (28). Similar to triglycerides, residual cholesterol is an important lipid metabolism index that has the potential to contribute more directly to insulin resistance. Although the mechanism by which residual cholesterol induces insulin resistance remains unclear, Wang et al. proposed that its proinflammatory features may promote aberrant insulin resistance by increasing inflammation and altering glucokinase activity (29). However, while individual lipid molecules have been more extensively studied as risk indicators for GDM, the role of NHHR in GDM remains underexplored. NHHR, as a composite indicator of multiple lipid parameters, may provide a more comprehensive approach to assessing the impact of lipid abnormalities. Reflecting more complex lipid metabolism dynamics, NHHR has garnered significant attention in medical research. A cross-sectional study using data from the 2005–2016 National Health and Nutrition Examination Survey found a linear dose–response relationship between NHHR and depression risk (P for non-linearity = 0.264), with this association remaining stable after sensitivity and subgroup analyses (13). Additionally, Lin et al. reported a significant correlation between NHHR and abdominal aortic aneurysms. Using PSM, they found that NHHR was a more robust indicator than individual lipid parameters, both before and after adjustment for confounders (21). Therefore, as an integrated marker of multiple lipid parameters, NHHR may offer a more comprehensive approach to assessing the relationship between dyslipidemia during pregnancy and GDM.

Although a positive correlation between NHHR and GDM is well-documented, the underlying mechanisms remain unclear. Drawing on existing physiological and metabolic studies, we have proposed several hypotheses to explore potential biological mechanisms. These mechanisms primarily involve the effects of hormonal changes, insulin resistance, and inflammatory responses. First, significant hormonal fluctuations occur during pregnancy, particularly elevated levels of placental hormones and estrogens, which lead to reduced insulin sensitivity in maternal tissues (30). Catalano et al., using the insulin-resistant clamp assay, demonstrated that these hormonal changes during pregnancy result in a 50–60% decrease in insulin sensitivity in both women with normal glucose tolerance and those with GDM (31). This insulin resistance primarily ensures that the fetus receives sufficient glucose for growth and development but may also increase the risk of GDM. Additionally, elevated estrogen levels can influence lipid metabolism, particularly by increasing hepatic non-HDL-C production and decreasing HDL-C clearance (32). Lin et al. found that NHHR levels were significantly higher in individuals with insulin resistance compared to those with normal insulin sensitivity (*p* < 0.05). Moreover, early pregnancy is often accompanied by increased levels of lipids such as total cholesterol and LDL-C, leading to elevated levels of free fatty acids in the bloodstream (33). Excess free fatty acids that are not metabolized through *β*-oxidation may accumulate over time, impairing insulin sensitivity (34). This set off a vicious cycle between lipid metabolism disorders and insulin resistance, further contributing to impaired glucose tolerance and the development of diabetes. Second, inflammation and oxidative stress play crucial roles in regulating immune dynamics in pregnant women. Previous studies have shown that pregnancy is a unique period for the regulation of inflammation and immune functions in women, with systematic changes in inflammatory and immune status (35). Studies have shown that tumor necrosis factor-alpha (TNF-*α*) and C-reactive protein levels at 24–28 weeks of gestation are strongly correlated with pregnancy-associated insulin resistance, making them potential inflammatory biomarkers for GDM management. Elevated levels of TNF-*α* are primarily produced by M1-type macrophages infiltrating adipose tissue, which inhibit insulin signaling through pathways, such as c-Jun N-terminal kinase and nuclear factor kappa B, and increase serine phosphorylation of insulin receptor substrate 1. Additionally, inflammatory factors such as TNF-α may further exacerbate lipid metabolism disorders and insulin resistance by directly affecting the expression and activity of lipid-synthesizing enzymes, such as by inhibiting the activity of lipoprotein lipase, which reduces fatty acid uptake and lipid synthesis (36). Meanwhile, elevated NHHR may suggest a dual state of imbalance between the accumulation of harmful lipoproteins and the absence of protective lipoproteins. This imbalance may stimulate the release of excess free fatty acids from adipose tissue into the circulation. These free fatty acids accumulate in peripheral tissues and disrupt the insulin signaling pathway through the activation of pathways such as protein kinase C (37) and JNK (38), leading to impaired glucose uptake and insulin resistance. Therefore, the relationship between the rise in NHHR during early and mid-gestation and the increased risk of GDM is closely associated with changes in hormonal levels, insulin resistance, and immune inflammation. Further investigation into these underlying mechanisms could offer opportunities for early intervention to reduce the prevalence of diabetes during pregnancy.

The primary strength of this study is the novel identification of mid-pregnancy NHHR as an independent predictor of GDM, supported by a detailed analysis of the correlation between the two. However, the study design has several limitations. First, due to its cross-sectional nature, this study could not establish a causal relationship between NHHR and GDM. Second, despite adjusting for various covariates, key variables such as body mass index (BMI), smoking status, and alcohol consumption history were not included due to an increased rate of missing data (over 20%). These data omissions could affect the completeness and reliability of the conclusions. Third, the limited sample size may lead to reduced statistical power, increased risk of selection bias, and difficulties in adequately adjusting for potential confounding variables. Moreover, the generalizability of the results to broader populations should be approached with caution. The smaller scale may also introduce a higher risk of publication bias and methodological constraints. Fourth, the study lacked data on the family history of diabetes. Additionally, we were unable to fully assess the dynamics of NHHR in early pregnancy to mid-pregnancy, as participants were unable to attend all scheduled follow-up visits. Furthermore, although this study confirmed a significant association between NHHR and GDM, the underlying biological mechanisms remain unclear. Therefore, future research should focus on the dynamic changes in NHHR throughout pregnancy and its impact on the risk of GDM to validate these preliminary findings and further elucidate the pathogenesis of GDM. Finally, while the results of this study indicate that NHHR has some value in the early prediction of gestational diabetes mellitus, its clinical discriminatory ability is limited. It needs to be evaluated in combination with other indices to improve its predictive accuracy.

## Conclusion

This study confirms that elevated NHHR levels are independently and positively correlated with the risk of developing GDM, highlighting their potential as a predictive biomarker for GDM. Abnormal NHHR levels can help identify individuals at high risk for GDM, allowing healthcare providers to plan and recommend early preventive lifestyle modifications. Elevated NHHR levels in early pregnancy to mid-pregnancy may serve as an effective biomarker for GDM screening.

## Data Availability

The raw data supporting the conclusions of this article will be made available by the authors, without undue reservation.

## Glossary

HDL-C: High-density lipoprotein cholesterol
non-HDL-C: Non-high-density lipoprotein cholesterol
NHHR: Non-high-density lipoprotein cholesterol-to-high-density lipoprotein cholesterol ratio
GDM: Gestational diabetes mellitus
LASSO: Least Absolute Shrinkage and Selection Operator
ROC: Receiver operating characteristic
T2DM: Type 2 diabetes mellitus
LDL-C: Low-density lipoprotein cholesterol
APTT: Activated partial thromboplastin time
PT: Prothrombin time
INR: International Normalized Ratio
TT: Fibrinogen, thrombin time
FT3: Free triiodothyronine
FT4: Free thyroxine
TSH: Thyroid-stimulating hormone
GGT: Gamma-glutamyltransferase
AUC: Area under the ROC curve
PSM: Propensity score matching
WBCs: White blood cells
RCS: Restricted cubic spline
TNF-*α*: Tumor necrosis factor-alpha
