# Supplementary material for: Association between non-high-density lipoprotein cholesterol-to-high-density lipoprotein cholesterol ratio and the risk of gestational diabetes: a retrospective study
Source: Front Nutr. 2025 Jul 3;12:1617225. doi: 10.3389/fnut.2025.1617225 (PMC12267022; doi:10.3389/fnut.2025.1617225)
Supplement: Supplementary file 1 [file Table_1.doc]

Supplementary table 1 GDMResults of LASSO regression coefficients for relevant important variables

| CoefName | coeff_min_lamda | coeff_se_lamda |
| --- | --- | --- |
| (Intercept) | -6.033379849 | -2.799164783 |
| Age | 0.173592848 | 0.120734852 |
| WBC | 0.031424923 | 0 |
| Blood platelet | 0.001532864 | 0 |
| Glutamine transpeptidase | 0.012430692 | 0 |
| Triglyceride | 0.090471568 | 0 |
| NHHR | 0.532244262 | 0.228317457 |
| APTT | -0.002070381 | 0 |
| FT3 | 0.045373431 | 0 |
