# Supplementary material for: Association between non-high-density lipoprotein cholesterol-to-high-density lipoprotein cholesterol ratio and the risk of gestational diabetes: a retrospective study
Source: Front Nutr. 2025 Jul 3;12:1617225. doi: 10.3389/fnut.2025.1617225 (PMC12267022; doi:10.3389/fnut.2025.1617225)
Supplement: Supplementary file 2 [file Table_2.doc]

Supplementary Table 2 Variance inflation factors for each variable

| VariableName | VIF |
| --- | --- |
| Age | 1.357339465 |
| Leucocyte | 1.205179024 |
| Blood platelet | 1.123667193 |
| Glutamine transpeptidase | 1.076538924 |
| Uric acid | 1.146987217 |
| Triglyceride | 1.338514934 |
| NHHR | 1.226266068 |
| INR | 1.058515089 |
| APTT | 1.031637896 |
| TT | 1.05754455 |
| FT3 | 1.076587665 |
| Number of times produced | 1.291295188 |
| Assisted reproduction | 1.032259898 |
